# Natural deep eutectic solvents-based green extraction of vanillin: optimization, purification, and bioactivity assessment

**DOI:** 10.3389/fnut.2023.1279552

**Published:** 2024-01-19

**Authors:** Lingxia Xu, Fakhra Liaqat, Mahammed Ilyas Khazi, Jianzhong Sun, Daochen Zhu

**Affiliations:** ^1^Biofuels Institute, School of Emergency Management, School of the Environment and Safety Engineering, Jiangsu University, Zhenjiang, China; ^2^Jiangsu Collaborative Innovation Center of Technology and Material of Water Treatment, Suzhou University of Science and Technology, Suzhou, China

**Keywords:** vanilla pod, lignin, deep eutectic solvents, antioxidant, antibacterial

## Abstract

The sustainable extraction of natural compounds has recently attracted significant attention. The extraction of high-quality natural vanillin in active form is crucial for its efficient use in various industries, but conventional solvents are not suitable for this purpose. The flammability, volatility, and toxicity of organic solvents can harm extraction personnel, and their waste liquid can cause environmental pollution. Natural deep eutectic solvents (NADES) are cost-effective, environmentally friendly, biodegradable, and non-toxic organic alternative to conventional solvents. In this study, 20 different NADES were tested for the sustainable extraction of natural vanillin. Among these, a DES system composed of choline chloride: 1,4-butanediol: lactic acid exhibited the highest extraction rate (15.9 mg/g). Employing response surface methodology (RSM), optimal extraction conditions were determined, yielding a vanillin content 18.5 mg/g with water content of 33.9%, extraction temperature of 64.6°C, extraction time of 32.3 min, and a solid-liquid ratio of 44.9 mg/mL. Subsequently, the optimized NADES system was then assessed for reusability in extracting vanillin from vanilla pods and kraft lignin over three cycles, retaining 43% of its extraction efficiency and demonstrating potential for waste reduction. Purification of vanillin was achieved through chromatography using a non-polar resin SP700, with ethanol as a desorption eluent and a feed solution pH of 4.0, resulting in the highest vanillin purity. HPLC and GC-MS analyses confirmed purity, while antioxidant activity assays (DPPH and ABTS) showcased significant antioxidant activity of the purified vanillin. Moreover, vanillin exhibited notable antimicrobial activity against a panel of food-borne bacteria. This study introduces an environmentally friendly approach to vanillin extraction highlights using NADES, emphasizing the potential for producing high-quality bioactive vanillin with reduced environmental impact. The applicability of NADES systems extends beyond vanillin, offering a versatile method for extracting diverse natural compounds.

## Graphical Abstract

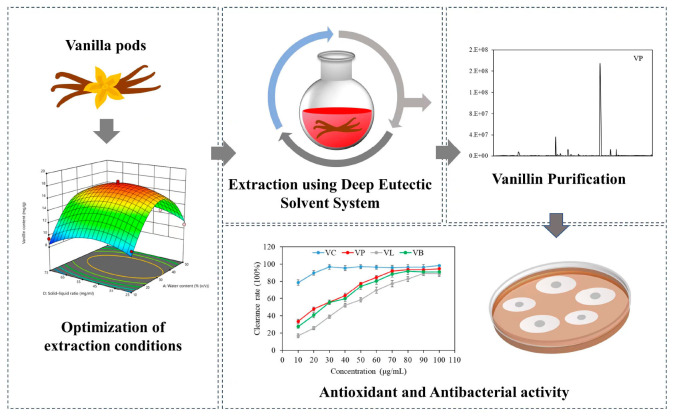

## 1 Introduction

Vanillin (4-hydroxy-3-methoxybenzaldehyde) is a popular flavoring ingredient with a strong vanilla aroma. It is the second most expensive flavoring agent in the world after saffron. It is extensively used in beverages, food, pharmaceuticals, cosmetics, and fragrances (1). Natural vanillin is a secondary metabolite of *Vanilla planifolia*, *Vanilla tahitensis*, and *Vanilla pompona*. Vanillin concentration in vanilla plant is approximately 2% of the dried weight of the vanilla beans after curing, which can contribute only 0.25% of the annual global consumption (>16,000 tons) of vanillin (2, 3). Growth of vanilla plants is slow, and their cultivation, pollination, harvesting, as well as hardening processing are challenging, which makes vanilla such an expensive source of vanillin (4, 5). Based on a vanillin yield of 20 g/kg dry weight, 50 kg of beans are required to yield 1 kg of vanillin, which needs 40,000 pollinated flowers. The cost of natural vanillin in 2018 reached that of silver, and the cured beans are sold for 50–80 $/kg. The current price of natural vanillin is about 25000 $/kg (6). Not only the cultivation but also the extraction of natural vanillin are laborious, and conventional extraction methods are expensive as well as time-consuming (7). Only natural vanillin is approved for food applications by most of the food safety authorities around the world (5). Therefore, the growing preference of consumers and the high demand for natural vanillin have brought attention to the necessity of developing cost-effective and environmentally benign vanillin synthesis and extraction techniques.

Deep eutectic solvents (DES), and Ionic liquids (ILs) have recently gained widespread recognition as environmentally friendly solvents and as substitutions for organic solvents. Their intriguing toxicological, physical, and bioactive properties can be finely adjusted by selecting the right precursors, such as viscosity, density, biodegradability, melting temperature, and solubility. ILs are salts that melt below 100°C, while DES are eutectic mixes made by hydrogen bonding between hydrogen bond acceptors (HBA) and donors (HBD). Nonetheless, some standard ILs and DES could be noxious based on the compounds used to make them. This could be the case with pyridinium, urea, imidazolium, and other substances. Like conventional DES, NADES are also transparent viscous liquids, with low melting point, formed by the hydrogen bonding of HBA and HBD. The HBAs consist of naturally occurring quaternary ammonium salts such as choline and betaine, and HBDs are natural products such as carboxylic acids, organic acids, amino acids, and glucose (8, 9). NADES are produced from non-toxic and natural sources, offering vistas for possible uses in the food and bio-product sectors (10). NADES are inexpensive, easy to produce, environmentally benign, biodegradable, and non-toxic organic molecules (11). In order to extract and isolate bioactive natural compounds from plant sources, DES and so-called NADES were previously used in a few studies (12–17). However, there have only been a few trials done, thus it is still unclear how effective NADES are in extracting natural products. Although a few NADES have been proposed as green solvents for natural vanillin extraction by one previous study (18), there is a need to evaluate their efficacy and potential in depth for extracting vanillin from vanilla beans as well as other natural sources, including lignin.

Apart from flavoring properties, vanillin is known for its antioxidant and antimicrobial activities, which allow it to be used as a substitute for synthetic chemical additives in food. However, some studies reported very low or no antioxidant activity of vanillin (19–21), whereas others reported that vanillin exhibited satisfactory scavenging activity (22, 23). Consequently, the findings on the antioxidant activity of vanillin are inconsistent, showing conflicting results, and a comparative study is essential to clarify the antioxidant activity of vanillin. Vanillin exhibited antimicrobial activity against bacteria, fungi, and yeasts (24). Vanillin has been applied to several foods as an antimicrobial against *food-borne pathogens* (25–27). However, there is no report regarding the antimicrobial activity of natural vanillin obtained from different sources. The present study addresses efforts to explore less costly NADES as green solvents for vanillin extraction. In order to develop a more affordable, environmentally friendly method of extracting vanillin, several NADES were tested in combination with water at various concentrations. Optimization of extraction rate has also been carried out using RSM, and the optimal extraction conditions were then employed to extract vanillin from lignin. This comprehensive optimization ensures that the highest possible yield of vanillin is obtained, reducing resource wastage, and maximizing productivity. We were curious to compare the biological activities of natural vanillin; therefore, the antioxidant and antibacterial activities of purified vanillin were also studied. The assessment of antioxidant and antimicrobial activities of the extracted vanillin provides valuable insights into its potential applications in various industries, such as food, pharmaceuticals, and cosmetics.

## 2 Materials and methods

### 2.1 Chemicals, reagents, and plant source

The *Vanilla planifolia* pods were purchased from Tmall, China sourced from Madagascar. Acetonitrile and phosphoric acid were HPLC grade and all other chemicals and reagents used in this investigation were of analytical grade and attained from Shanghai Macklin Biochemical Co., Ltd., Shanghai, China.

### 2.2 Natural deep eutectic solvent system preparation

To compare the vanillin extractability, 20 different NADES systems were prepared in certain molar ratios (Table 1). Heating-stirring method was used to prepare NADES and the components of each system were mixed and stirred at 400 rpm and heated at 80°C until a stable and clear liquid was formed (2–3 h) (28). To adjust the viscosity, 10% (v/v) deionized water was added to several NADES solutions.

**TABLE 1:** Different NADES systems used to extract vanillin.

Name	Combination	Molar ratio
DES1	L-Proline: glucose	1:1
DES2	L-Proline: malic acid	1:1
DES3	L-Proline: sucrose	2:5
DES4	L-Proline: lactic acid	2:5
DES5	Choline chloride: DL-malic acid	1:1
DES6	Choline chloride: lactic acid	1:1
DES7	Choline chloride: urea	1:2
DES8	Choline chloride: urea	1:5
DES9	Choline chloride: urea: lactic acid	1:1:1
DES10	Choline chloride: malic acid: xylitol	1:1:1
DES11	Choline chloride: malic acid: proline	1:1:1
DES12	Choline chloride: D fructose	1:1
DES13	Betaine: glycerol	1:2
DES14	Betaine: citric acid	1:1
DES15	Betaine: malic acid	1:1
DES16	Betaine: malic acid: glucose	1:1:1
DES17	Choline chloride: 1,4 butanediol	1:5
DES18	Choline chloride: 1,4-butanediol: lactic acid	1:1:1
DES19	Choline chloride: ethylene glycol	1:2
DES20	Choline chloride: glycerol	1:1

### 2.3 Extraction of vanillin from vanilla pods

Vanilla pods were naturally dried, cut into 2–3 cm pieces with scissors and moisture content was measured by oven drying testing. When the moisture content was between 1 and 3%, vanilla pods were ground to fine powder using Shanghai Xinuo portable high-speed grinder (DFT-100) with a size of 60 mesh. NADES-based ultrasound-assisted extraction was carried out by adding 50 mg dried vanilla pods powder into 2 ml of each NADES solution. Ultrasonic extraction was performed at a frequency of 25 kHz at 100 W for 20 min. The supernatant was collected after the suspension was centrifuged at 12000 rpm for 10 min. Supernatant was diluted with deionized water and filtered through 2.5 μ membrane filter prior to HPLC analysis. The vanillin was identified using an HPLC system (Shimadzu LC-6AD, Kyoto, Japan) supplied with a Shimadzu ODS-3 C18 analytic column (4.6 mm × 250 mm, 5 μm) and SPD-M20A UV-VIS/Photodiode Array (PDA) detector. Mobile phase was 0.1% acetonitrile and phosphoric acid (1:1), in isocratic mode, detection wavelength was 280 nm, sample injection volume was 20 μl and column temperature was 30°C. Extraction yields (Ey) were calculated using the formula given below:

Ey=Cf × Vs

Where Cf is the amount of vanillin determined by HPLC analysis to be present in DES, and Vs is the volume of the diluted solution. Vanillin standard (V1104, Sigma) was used to draw calibration curve for vanillin quantification.

### 2.4 Experimental design for optimization of vanillin extraction rate

Based on maximum extraction efficiency, one best NADES was selected for the optimization experiments. To reveal the influence of various parameters, the optimum values for the independent variables (Table 2), including water content in NADES (A), extraction time (B), extraction temperature (C), and solid-liquid ratio (D), were determined by using RSM and Box-Behnken design (BBD) at three levels (−1, 0, +1). All the experimentations were carried out in triplicates and data are presented as means ± standard error (SD).

**TABLE 2:** Four-factor BBD their units and levels.

Factors		Units	Level 1 (−1)	Level 2 (0)	Level 3 (1)
A	Water content	% (v/v)	10	30	50
B	Time	min	20	40	60
C	Temperature	°C	30	50	70
D	Solid–liquid ratio	mg/ml	25	50	75

### 2.5 Extraction of vanillin from lignin and recyclability of NADES

Best NADES and optimal extraction conditions were employed to extract vanillin from kraft lignin using ultrasound-assisted extraction method as described in section “2.4 Experimental design for optimization of vanillin extraction rate.” Three NADES extraction cycles were conducted to evaluate the extraction efficiency of reusing NADES solvent. Using a vortex shaker, vanillin was completely dissolved in 2 ml of ethyl acetate for 5 min. Phase separation was achieved by centrifuging the mixture for 10 min at 4500 rpm and the top ethyl acetate phase was removed. To regenerate the unreacted dissolved lignin, isopropanol was employed as an anti-solvent following the previous method (29).

### 2.6 Purification of vanillin

Vanillin extracted from vanilla plant pods by NADES was purified by adsorption chromatography using SP700 (Mitsubishi Chemical) non-polar resin by the method previously described (30). To select the best solvent for purification water and ethanol were individually tested and extraction yield was calculated. Different pH values were also tested to select the optimal pH for vanillin purification. Vanillin from lignin was also purified using optimal solvent and pH by SP700 resin. Microbial vanillin was synthesized by lignin degrading bacteria *Bacillus ligniniphilus* L1 using the method described in our previous study (31). Microbial vanillin was extracted from the fermentation broth by previously described method (32) and purified by employing three molecular weight cut-offs of 50, 5, and 1 kDa tubular ceramic membranes in a membrane fractionation process followed by chromatography using SP700 resin. Purified samples were analyzed by HPLC method described in section “2.4 Experimental design for optimization of vanillin extraction rate” and also identified by GC-MS analysis according the method reported in our previous study (31).

### 2.7 Antioxidant activity

2,2′-azino-bis(3-ethylbenzothiazoline-6-sulfonic acid) (ABTS) and 2,2-Diphenyl-1-picrylhydrazyl (DPPH) assays were carried out to test the antioxidant activity of pure vanillin extracted from diverse natural sources.

**DPPH assay:** DPPH assay was carried out by the method reported by Pedan et al. (33). Various concentrations of vanillin extract (10–100 μg/mL) were used to measure the antioxidant activity. The absorbance was measured at 517 nm using equivolume mixture of methanol and DPPH as blank solution. Vitamin C (VC) solution in methanol was used as a standard antioxidant. Each sample was measured three times. DPPH free radical scavenging activity was calculated according to the following equation:


DPPHradicalscavengingactivity(%)=[(Ao-Ai)/Ao]×100,

where Ai is the absorbance value of the samples or the standard compounds and Ao is the absorbance value of the blank.

**ABTS assay.** ABTS assay was carried out by the method reported by Pedan et al. (33). Equal quantities of 2.45 mM potassium persulfate and 7 mM ABTS were combined to formulate a solution of ABTS radical cations (ABTS⋅^+^) and solution was incubated for 12 to 14 h at 25°C in the dark. Then 50 μL of various concentrations (10–100 μg/mL) of vanillin was mixed with 150 μL ABTS⋅^+^ and incubated in the dark for 30 min. VC was used as a standard antioxidant and absorbance was measured at 734 nm. 50 μL methanol was added into ABTS⋅^+^ solution instead of sample to serve as blank. Each sample was measured three times. ABTS free radical scavenging activity was calculated according to the following formula:


ABTSradicalscavengingactivity(%)=[(Ao-Ai)/Ao]×100,

where Ai is the absorbance value of the samples or the standard compounds and Ao is the absorbance value of the blank. The antioxidant activity was represented as an IC50 value using the concentration-dependent linear regression curve for DPPH and ABTS. The IC50 value in μM is the concentration at which DPPH or ABTS radicals are scavenged by 50%.

### 2.8 Antibacterial activity

Purified vanillin was tested by agar disk diffusion assay for its antibacterial properties against predominant food borne pathogens including *Escherichia coli* (CMCC44102), *Salmonella paratyphi* (CMCC50094), *Shigella sonnei* (CMCC51105), *Vibrio parahaemolytieus* (ATCC17802) *Staphyloccocus aureus* (CMCC26003), and *Listeria monocytogenes* (ATCC19115). Bacterial suspensions containing 10^6^ CFU/mL were spread uniformly on Mueller–Hinton agar with a sterile cotton swab. Disks of 6 mm × 1 mm size were employed on the plates with sterile forceps. 100 μL of vanillin solution in ethanol with a concentration of 5 mg/ml (pre-determined by calculating the minimum inhibitory concentrations (MICs) of vanillin standard against each tested bacterial strain) was added to disks and the plates were retained at 4°C for 30 min to allow the extract to pre-diffusion into the agar. The plates were then incubated at 37°C for 18 h. Ethanol was used as the negative control.

### 2.9 Statistical analysis

One-way analysis of variance (ANOVA), was used for statistical analysis followed by *post hoc* Tukey’s test by SPSS 19.0. Resulting *p*-value < 0.05 were deliberated as significant. Data are presented as means ± standard error (SD) in text, tables, and figures.

## 3 Results and discussion

### 3.1 Extraction of vanillin with NADES

**FIGURE 1:**
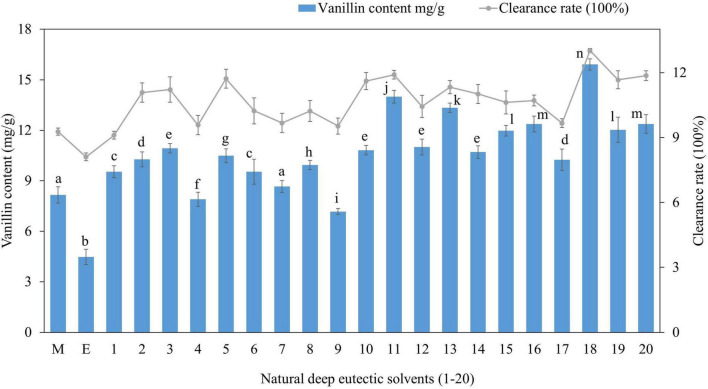
Vanillin content extracted from vanilla plant using 20 different deep NADES and antioxidant activity of crude extracts measured by DPPH assay. The data is presented as mean ± SD (*n* = 3). Extraction efficiencies that differ significantly (*p* < 0.05) are indicated with different alphabets.

For vanillin extraction from vanilla pods, 20 distinct NADES types representing various NADES classes were developed from three groups of HBAs (Choline chloride, betaine, and L-proline) and four types of HBDs (carboxylic acids, alcohols, amides, and sugars). To identify the most effective NADES system, the total vanillin content (mg/g) recovered was taken into consideration, and the results are summarized in Figure 1. There were significant differences in the extractability of vanillin using 20 different NADES, resulting in varying yields. Vanillin was highly extractable in choline chloride: 1,4-butanediol: lactic acid (NADES-18) which produced the maximum extraction yield (15.9 mg/g), whereas choline chloride: urea: lactic acid (NADES-9) yielded the minimum amount of vanillin (7.2 mg/g). L-proline and betaine-based NADES systems yielded a slightly lower amount and were clearly less efficient. Methanol and ethanol were also tested for vanillin extraction, and their yields were substantially lower (*p* < 0.05) than those of the majority of the investigated NADES, most likely owing to vanillin’s stronger ability to establish hydrogen bonds with these NADES. Vanillin is a hydrophobic compound with methoxy and hydroxy groups in its 3- and 4-positions. Choline chloride-based NADES are hydrophilic and have higher polarity than water, which can be adjusted by changing the water content in NADES formulation. They present high selectivity and solubility for phenolic compounds due to their strong hydrogen bonds with target compounds. Increasing the number of hydroxyl groups of HBD molecules surrounding the negatively charged chloride anion of choline chloride can decrease the polarity of solvent (34). 1,4-butanediol has two hydroxyl groups and stronger hydrogen bonding, thus showed better extraction efficiency with choline chloride in DES18, while urea lacks a hydroxyl group and showed the lowest extraction efficiency in DES9. Previous study reported that the excellent dissolution properties of DES solvents are due to the formation of hydrogen bonds (35). It has also been reported that NADES composed of choline chloride and urea are more hydrophilic and ideal for the extraction and enrichment of hydrophilic compounds (36). Liu et al. (37) found that among the 7 prepared NADES, choline chloride and lactic acid (molar ratio 1:2) combined with ultrasound extraction showed the best effect (3.53 ± 0.03 g GAE/100 g) on the extraction of total polyphenols from *Cosmos sulphureus.* The total polyphenolic content was 21% higher than the ultrasound assisted ethanol extraction rate (2.92 ± 0.07 g GAE/100 g). This might be attributed to the polarity of the carboxyl group of lactic acid in NADES which is closer to the polarity of the polyphenol compound, resulting in better dissolution of the target polyphenol (vanillin). The extraction efficiencies of sugar-based NADES (DES1, DES3, DES12, DES16) were not very high (Figure 1) due to their high viscosities which can hinder mass transfer between the solvent and the product, potentially affecting the extraction efficiency (38). Vanillin extraction efficiency decreased when sugars were used as hydrogen bond donors in NEDES due to higher viscosity, which hinders mass transfer and decreases extraction efficiency. In the presence of alcohol and organic acids, viscosities are low, resulting in higher extraction. In general, the composition (group of HBA and HBD, water content, and molar ratio) and physicochemical characteristics (viscosity, surface tension, pH, and polarity) of the DES components determine the extraction capability of NADES for bioactive chemicals from plants (39). Due to the van der Waal forces, hydrogen bonds, and electrostatic interactions between the components of DES, they have greater viscosities than traditional solvents, which leads to slow mass transfer and limits their utility in the extraction of naturally occurring bioactive chemicals (17). Therefore, additional water was added to some NADES to adjust the viscosity.

In addition, we also investigated the antioxidant activity of crude vanillin extracted by different NADES (Figure 1) and results revealed a correlation between vanillin content and DPPH free radical scavenging activity since NADES-18 yielding the highest content showed the highest antioxidant activity and NADES with low extraction efficiency displayed low antioxidant activity; accordingly, NADES-18 was chosen for optimization studies. Vanillin extracted by ethanol exhibited significantly lower (*p* < 0.05) antioxidant activity compared to NADES extracted vanillin. These findings showed that NADES not only exhibited superior extractability of vanillin but also maintained its greater antioxidant activity. This can be attributed to intermolecular interactions between DES and natural compounds, which can reduce their oxidative degradation because they slow down solute molecule mobility, leading to reduced oxygen contact (40).

### 3.2 Optimization of extraction conditions

A previous study showed that parameters like water content and temperature can greatly influence the viscosity of DES (18). Therefore, in present study, we selected four independent variables, including extraction temperature, solid–liquid ratio, water content in NADES, and extraction time. Box-Behnken surface model was employed to optimize the extraction of vanillin for NADES-18 using three levels of each variable. The vanillin content (mg/g) was used as a response to assess the extraction efficiency of the model. To avoid systematic errors, the experiments were carried out in a random manner and the results of experiments are given in Table 3. A second-order polynomial equation was used to describe the predicted model, and regression analysis was performed on the experimental data. The regression equation for the variables and the responses in terms of the coded levels is given below:


Y=17.86+1.03A-0.0977B+1.06C-0.3950D

        - 0.0175AB+0.7650AC+0.1750AD-0.7433BC

$$+0.9250BD+0.1750CD-5.90A^{2}-1.27B^{2}-0.6498C^{2}-1.94D^{2}$$

**TABLE 3:** Box-Behnken design for the optimization of independent variables of NADES-based vanillin extraction and the resulted response values.

Run	A	B	C	D	Vanillin content (mg/g)
1	0	0	0	−1	14.82
2	0	0	0	1	15.27
3	0	1	1	0	14.04
4	0	0	0	0	17.8
5	0	0	0	0	17.95
6	0	0	−1	1	13.82
7	−1	−1	0	0	9.2
8	0	−1	0	1	13.71
9	1	0	0	1	10.39
10	0	1	1	0	17.51
11	0	0	0	0	18.36
12	0	−1	−1	0	14.94
13	1	0	1	0	15.68
14	−1	1	0	0	10.09
15	1	1	0	0	11.92
16	0	1	−1	0	15.46
17	0	0	0	0	18.1
18	0	−1	0	−1	16.35
19	0	0	−1	−1	15.72
20	1	−1	0	0	11.1
21	−1	0	1	0	10.5
22	−1	0	0	1	9.39
23	−1	0	0	−1	10.39
24	0	0	0	0	17.99
25	−1	0	−1	0	8.31
26	1	0	−1	0	10.43
27	0	0	1	1	15.41
28	0	0	1	−1	16.61
29	1	0	0	−1	10.69

A: Water content, B: Time, C: Temperature, D: Solid–liquid ratio.

**FIGURE 2:**
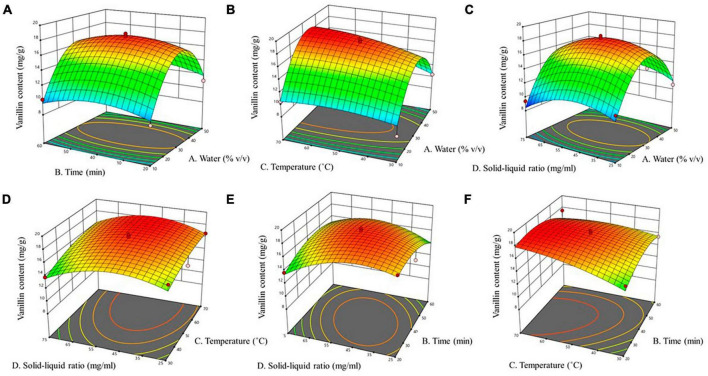
Effect of interaction between various factors on vanillin content. **(A)** water content and time, **(B)** water content and temperature, **(C)** water content and solid-liquid ratio, **(D)** temperature and solid-liquid ratio, **(E)** time and solid-liquid ratio, **(F)** time and temperature.

A represents water content in NADES; B represents time; C represents temperature; and D represents solid to liquid ratio. The square of the correlation coefficient (R2), was used to determine the model’s efficiency, and ANOVA with a 95% confidence level was used to evaluate lack of fit. The coefficient of determination (R^2^) was 0.932, indicating that 93% of the variance of the response is explained by the variance of the independent variables. Since the lack-of-fit model’s *F*-value was non-significant (Table 4), the adequacy of model was validated. The low *p*-value (<0.0001, highly significant) and high *F*-value (13.66) demonstrate the ideality of quadratic polynomial fitting equation, and high significance of model, indicating the reliability of the experimental method. The lack of fit (*p* = 0.4457 > 0.05) provides that the regression model has a good fit, indicating the results are not significant compared to pure error. We employed three-dimensional (3D) response surface plots (Figure 2) for the elucidation of interactions amongst the four independent variables on the dependent variable vanillin extraction, which demonstrates that the yield of vanillin extraction is certainly correlated to the main variable. In the BBD, the extraction time, and the solid-liquid ratio both revealed a non-significant influence (*p* > 0.05), while the water content in the NADES solution and extraction temperature showed statistically significant (*p* < 0.05) effects on vanillin extraction. These outcomes revealed by 3D plots are analogous to those found by ANOVA, where the water content and temperature exhibited a significant effect on the vanillin content. In the case of water content, the extraction yield was greatly enhanced up to 34% water content and then decreased rapidly beyond that. The highest vanillin extraction yield was recorded at 33.9% water content, as illustrated in Figure 2. The contour graphs show elliptical shape and steep response faces, indicating significant interaction between water content, time, and temperature (Figures 2A–C). However, the interaction between time, temperature, and solid-liquid ratio is not significant, especially between the solid-liquid ratio and time (Figures 2D, F). RSM contour and 3D surface plots indicate that the rate of vanillin extraction increases with increasing water content and extraction temperature, but decreases when these factors exceed a certain threshold. Water might be playing a role in improving the polarizability while reducing the HBA basicity (41). Water content is a crucial factor that can be employed to change the properties of NADES and increase their specific extractability for target chemicals, since water content can define the polarity and viscosity of NADES. However, very high-water content can inversely affect the extraction efficiency of NADES. As reported previously, when 50% water content was used, the interactions due to hydrogen bonding between the DES ingredients disappeared, reducing the interfaces between the solvent and the desired compounds and resulting in poor extraction efficiency (42).

**TABLE 4:** Variance analysis of the quadratic model for vanillin by deep eutectic solvent extraction.

Source	Sum of squares	df	Mean square	*F*-value	*p*-value	
Model	266.28	14	19.02	13.66	<0.0001	significant
A-A	12.67	1	12.67	9.10	0.0092	
B-B	0.0806	1	0.0806	0.0579	0.8134	
C-C	11.55	1	11.55	8.30	0.0121	
D-D	1.56	1	1.56	1.12	0.3077	
AB	0.0012	1	0.0012	0.0009	0.9768	
AC	2.34	1	2.34	1.68	0.2157	
AD	0.1225	1	0.1225	0.0880	0.7711	
BC	1.61	1	1.61	1.16	0.2157	
BD	1.43	1	1.43	1.02	0.3286	
CD	0.1225	1	0.1225	0.0880	0.7711	
A^2^	227.49	1	227.49	163.43	<0.0001	
B^2^	8.89	1	8.89	6.39	0.0241	
C^2^	2.54	1	2.54	1.82	0.1983	
D^2^	21.08	1	21.08	15.14	0.0016	
**Residual**	19.49	14	1.39			
Lack of fit	13.29	9	1.48	1.19	0.4457	not significant
Pure error	6.19	5	1.24			
**Cor total**	285.77	28				

The viscosities of NADES were also reported to be quite temperature-sensitive. Temperature can influence the solubility and diffusion rate of solvents, thereby can decrease viscosity, and exert a positive effect on natural compounds extractability (43). In the present study, extraction temperature positively affected vanillin extraction between the ranges of 30–65°C. However, temperatures above 65 decreased the extractability of vanillin, attributed to vanillin’s heat-induced decomposition (Figure 2). Time and the solid-liquid ratio had less of an impact on the extraction of vanillin than the other two variables (Figure 2). RSM-determined optimal conditions for NADES vanillin extraction are given in Table 5. Under these conditions, an extraction yield of 18.5 mg/g was confirmed in a verification experiment. A previous study on vanillin extraction using NADES verifies our results where they reported the influence of water content on the NADES extractability of vanillin (18).

**TABLE 5:** Optimal values of the four variables that yields the maximum vanillin content in RSM.

A	B	C	D	Vanillin content	Desirability	
33.9	32.3	64.6	44.9	18.5	1.000	Selected

### 3.3 Extraction of vanillin from lignin and recyclability of NADES

**FIGURE 3:**
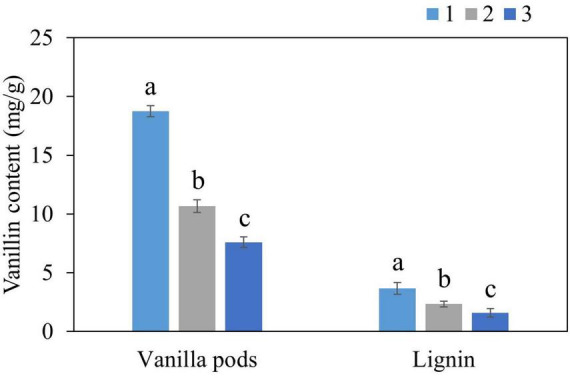
Three cycles of vanillin extraction from vanilla plant, and lignin using NADES-18 under optimal extraction conditions.

Optimal extraction conditions were employed to extract vanillin from kraft lignin and vanilla pods. NADES were recovered after the extraction step and three cycles of extraction were performed in order to test the reusability of NADES (Figure 3). 10.7 mg/g vanillin was extracted from vanilla pods in the second cycle and 7.6 mg/g in the third cycle, whereas 2.3 mg/g vanillin was obtained from lignin in the second cycle and 1.6 mg/g in the third cycle. These results clearly showed that NADES system can be a cost-effective and sustainable alternative for natural vanillin extraction since it is reusable, and even after the third cycle, it was able to present 43% extractability. The capacity of NADES to be reused may aid in overcoming the difficulties associated with recovery and recycling, thereby increasing the feasibility and efficacy of the extraction procedures. Some previous studies with DES extraction of natural compounds also proved the recyclability and reusability of these solvents over conventional solvents, which supports our findings (44, 45).

### 3.4 Purification of vanillin

In order to test the biological activities of natural vanillin extracted from plants and lignin using NADES, we further purified the vanillin by membrane fractionation followed by chromatography with SP700 resin. Green solvents offer promising extraction capabilities, but their low volatility and compatibility with conventional purification techniques require specialized methods for efficient solvent removal (1). In recent years, the use of polymeric resins to recover phenolic chemicals from various media has gained attention. Given that these resins have a high capability for adsorption, quick recovery of the phenolic compounds, low cost, and straightforward regeneration, their usage in industrial-scale processes is quite promising (30). These resins are food-grade, making them suitable for use in the pharmaceutical and food industries. Additionally, the hydrophobic and hydrophilic groups present in these resins aid in the adsorption of phenolic chemicals (30). Various studies utilizing complex solutions have shown that these resins have a great adsorption aptitude for a wide range of phenolic chemicals and are easily recoverable from aqueous or organic solvents. Studies have also shown that vanillin and other phenolic compounds have greater resin capacities in non-polar resins compared to those using ion-exchange resins (46, 47).

**FIGURE 4:**
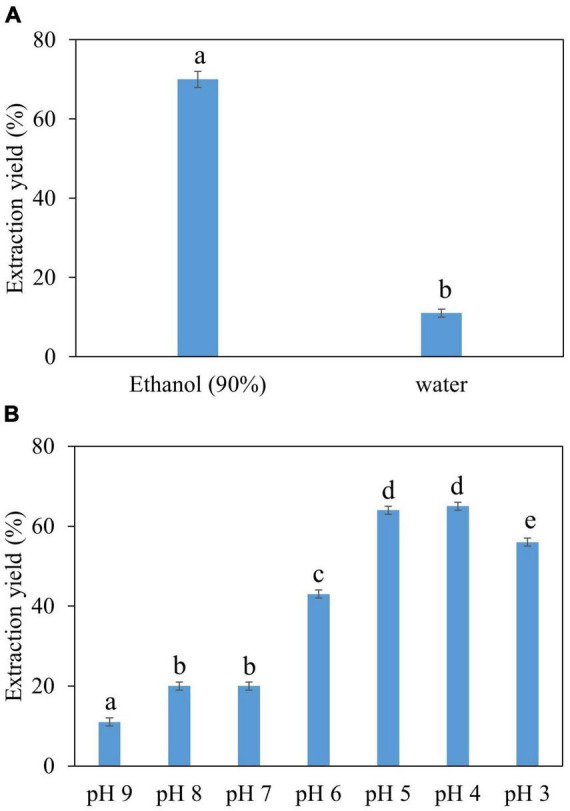
Optimization of vanillin purification conditions, **(A)** selection of optimal solvent, **(B)** selection of optimal pH.

**FIGURE 5:**
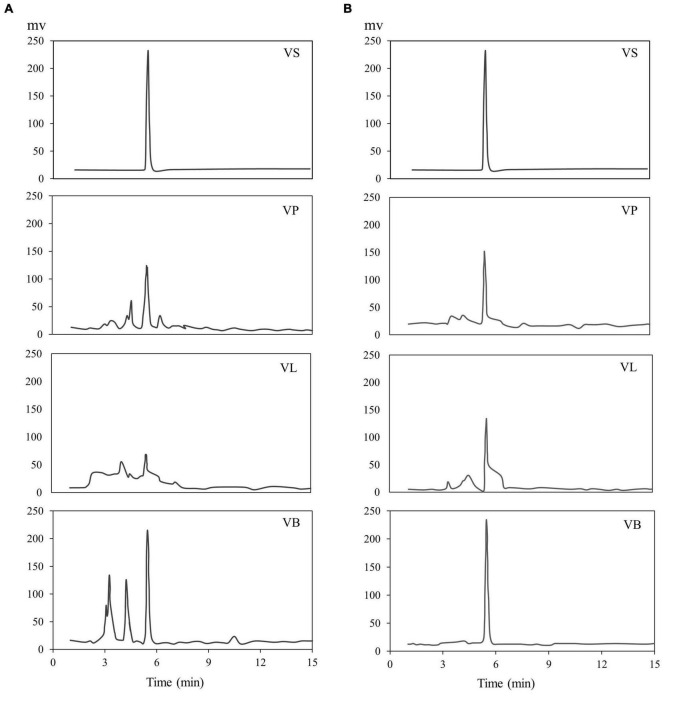
HPLC chromatogram of vanillin, **(A)** after extraction and **(B)** after purification. VS; vanillin standard, VP; vanillin from vanilla pod, VB; vanillin from bacterial fermentation broth, VL; vanillin from lignin.

**FIGURE 6:**
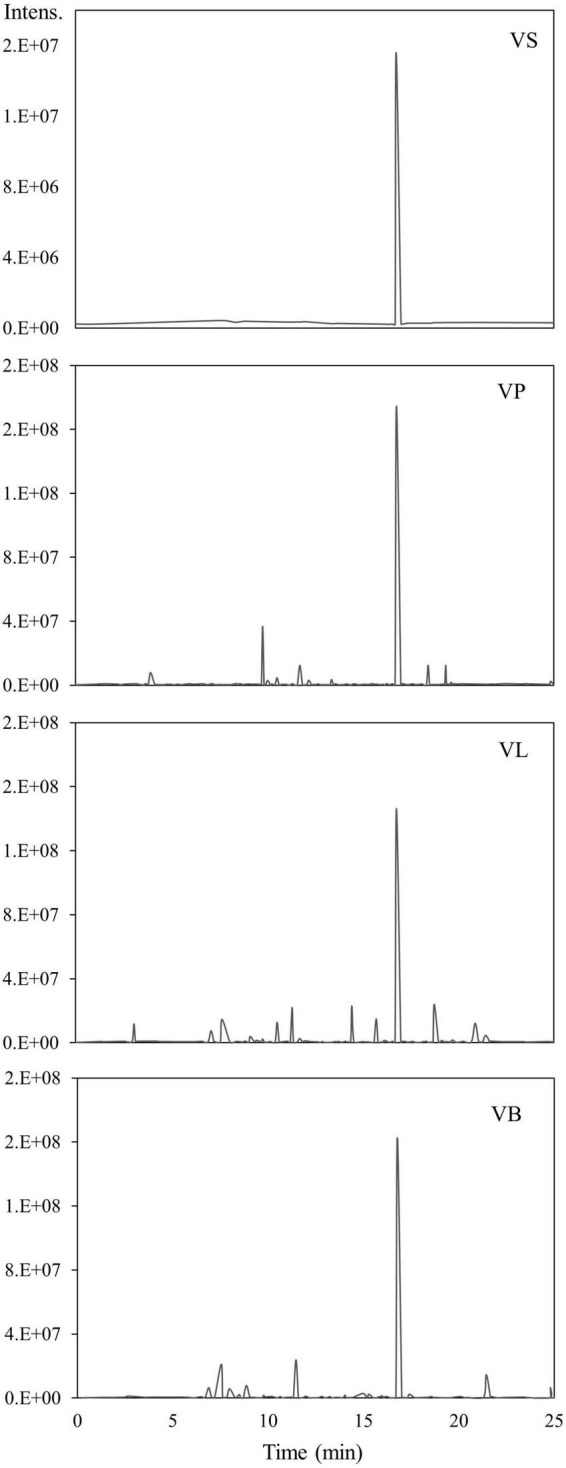
GC-MS analysis of purified vanillin. VS; vanillin standard, VP; vanillin from vanilla pod, VB; vanillin from bacterial fermentation broth, VL; vanillin from lignin.

Water and ethanol were tested as desorption solvents for chromatographic purification. In previous studies, both water and ethanol were tested as eluents of desorption solvents in chromatographic purifications. For instance, Forss et al. (48) used a strong cation-exchange resin with water as an eluent to purify the phenolic compounds, including vanillin, from oxidized spent sulfite liquor containing lignin. While Gomes and Rodrigues (49) used water and ethanol for desorption. In the present study, 90% ethanol showed significantly greater elution efficacy compared to water because vanillin solubility in water is very low compared to ethanol. Previous studies demonstrated that the phenolic monomers may be easily eluted with low bed volumes of organic solvents (30, 47, 50). Additionally, the pH value of each feed solution was corrected to 3–9 before feeding into the SP700 resin bed, and various pH values were evaluated (Figure 4) in the quest for the highest vanillin yield. Acidic pH 4 and 5 yielded better extraction 65 and 64% of vanillin compared to basic pH. A previous study by Wang et al. (51) verifies our finding where vanillin and syringaldehyde were effectively recovered from spent liquor using non-polar resin, with better adsorption capabilities at lower pH. Vanillin purified from all three natural sources was analyzed and quantified by HPLC (Figure 5) and further confirmed by GC-MS analysis using a known standard as a control (Figure 6).

### 3.5 Antioxidant and antimicrobial activity of vanillin

**FIGURE 7:**
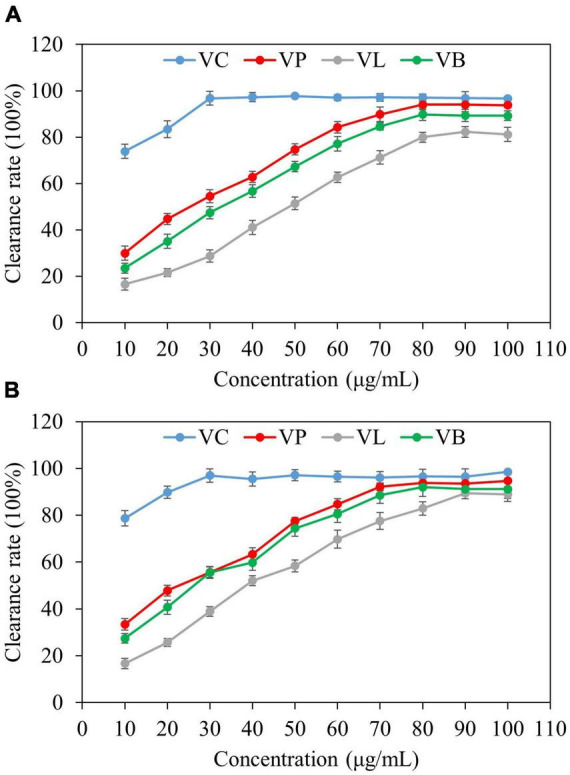
Antioxidant activity of purified vanillin determined by two different assays. VC; Vitamin C, VP; vanillin from vanilla pod, VB; vanillin from bacterial fermentation broth, and VL; vanillin from lignin. **(A)** DPPH radical scavenging activity, **(B)** ABTS radical scavenging activity.

Antioxidant activities of vanillin from vanilla pod (VP), vanillin from bacterial fermentation broth (VB), and vanillin from lignin (VL) were determined by two different anti-oxidant assays (DPPH and ABTS) and the results were compared (Figure 7). All types of vanillin showed antioxidant activity in both type of assays. Activity was concentration-dependent up to 80 μg/ml. However, the antioxidant capacity of vanillin displayed a threshold point at a certain concentration, as shown by the fact that subsequent concentration increase did not result in a rise in the antioxidant activity. VC was used as a standard antioxidant (positive control), and the antioxidant activity of NADES-extracted vanillin was comparable to that of VC at a concentration above 50 μg/ml. Studies show that DES extraction of natural products not only offers high efficiency and yield, but also significantly improving the extract’s ability to scavenge DPPH and ABTS free radicals (52). NADES, due to their unique physical properties and non-covalent interactions with target compounds, can efficiently lyse plant cell walls and extract various bioactive substances (53). This study utilized ultrasonic-assisted extraction, which accelerates the dissolution of the target substance, resulting in a higher extraction rate. A comparative study demonstrated the significant synergistic effect of ultrasonication over other methods in terms of bioactive components and antioxidant activities (54). In a previous study by Dalmolin et al. (55), vanillin showed concentration-dependent scavenging activity, and similar to our findings, the two highest concentrations of vanillin (31.25 and 15.625 g/mL) showed roughly the similar activity. In another study, vanillin showed greater ABTS⋅^+^-scavenging activity than Trolox and ascorbic acid, but it exhibited no antioxidant activity in the DPPH and galvinoxyl radical-scavenging tests (56). In the ABTS⋅^+^-scavenging assay, vanillin reacted with radicals by a self-dimerization reaction. The vanillin’s potent activity was a consequence of the high reaction stoichiometry against ABTS⋅^+^ radicals, which was facilitated by dimerization (56).

The antimicrobial activity of vanillin standard (VS), VP, VB, and VL was tested against various food-borne pathogens by agar disk diffusion assay. To determine the concentration of vanillin to be incorporated in the disks, the MIC of VS was first calculated against all the test strains (Supplementary Table 1) and based on the results, the concentration was selected for all types of natural vanillin. The antibacterial activity in terms of zone of inhibition is presented in Table 6. Among various types of natural vanillin, VP showed greater inhibitory effects against all tested strains whereas VL showed the lowest amount of antimicrobial activity. All types of natural vanillin displayed antibacterial activity against both gram-negative and gram-positive pathogens, except VL, which showed no inhibitory effect against *S. aureus*. VB also showed satisfactory antibacterial effects against all test strains. To the best of our knowledge, no previous report has described the antimicrobial activity of natural vanillin. In previous studies on the antimicrobial activities of vanillin, commercial vanillin was used, for instance, in the studies by Cava-Roda et al. (26) and Moon et al. (25). Furthermore, in some studies, vanillin was used in combination with other antimicrobial products, such as essential oils (27) and potassium sorbate (57). In a recent study, synthetic vanillin (5 mg/mL) exhibited robust antimicrobial activity against gram-negative as well as gram-positive bacteria, with zones of inhibition ranging from 11.67 to 12.75 (58) which is comparable to our results.

**TABLE 6:** Antimicrobial activity (zone of growth inhibition in mm) of vanillin against the bacterial strains tested by disk-diffusion method.

Vanillin type	VS	VP	VB	VL
*E. coli*	14.5 ± 0.2	14.0 ± 0.3	13.4 ± 0.4	10.2 ± 0.2
*S. paratyphi*	10.6 ± 0.2	8.9 + 0.3	8.7 ± 0.2	7.6 ± 0.4
*S. sonnei*	13.4 ± 0.2	12.0 ± 0.1	10.3 ± 0.1	8.1 ± 0.3
*V. parahaemolytieus*	13.4 ± 0.4	13.2 ± 0.3	12.2 ± 0.1	9.8 ± 0.3
*L. monocytogenes*	9.4 ± 0.4	9.3 ± 0.3	8.9 + 0.3	7.1 + 0.1
*S. aureus*	10.3 ± 0.1	9.8 ± 0.4	9.0 ± 0.2	-

VS, vanillin standard; VP, vanillin from vanilla pod; VB, vanillin from bacterial fermentation broth; VL, vanillin from lignin. Concentration of the vanillin for each bacterial strain was selected based on MIC experiment results (Table1S). -; no zone of inhibition.

## 4 Conclusion

This research successfully developed a sustainable extraction method for of natural vanillin using a novel NADES system. Employing RSM, optimal extraction conditions were determined, enhancing the efficiency of vanillin extraction. Notably, the optimized NADES system exhibited remarkable reusability, maintaining 43% of its extraction efficiency after three cycles, thereby minimizing waste and promoting environmentally sustainability. Subsequent purification of the extracted vanillin was achieved through chromatography employing a non-polar resin, leading to the attainment of a satisfactory level of purify. The purified vanillin displayed noteworthy antioxidant properties, indicating its potential as a valuable natural antioxidant additive. Furthermore, the purified vanillin exhibited substantial antimicrobial activity against predominant food-borne pathogens, indicating its possible application in food preservation and safety. This study marks a significant advancement in the field of sustainable vanillin extraction, contributing to eco-friendly practices. Moreover, the potential utilization of vanillin as an antioxidant and antimicrobial agent in diverse applications is underscored. This eco-friendly extraction approach proposed here holds promise for enhancing the sustainability and marketability of vanillin, while also providing a template for the extraction of other natural compounds. Although NADES come from natural compounds, there is currently limited research on the safety testing of their extracted products, and further in-depth research is needed. Research on the specific mechanism of the high stability and activity of NADES extracts is limited compared to traditional solvents, requiring further strengthening. This study lacks *in vivo* research on vanillin’s antimicrobial and antioxidant properties, which are crucial for its potential applications in antisepsis, cosmetics, and the food industry. Additionally, the current research is mostly limited to laboratory scale and requires further expansion of clinical trials to achieve industrialization.

## Data availability statement

The original contributions presented in this study are included in the article/Supplementary material, further inquiries can be directed to the corresponding authors.

## Author contributions

LX: Formal analysis, Investigation, Methodology, Writing – original draft. FL: Data curation, Formal analysis, Investigation, Methodology, Validation, Writing – original draft. MK: Data curation, Methodology, Software, Validation, Writing – review and editing. JS: Funding acquisition, Resources, Supervision, Writing – review and editing. DZ: Conceptualization, Funding acquisition, Methodology, Project administration, Resources, Supervision, Writing – review and editing.
