# Supplementary material for: Natural deep eutectic solvents-based green extraction of vanillin: optimization, purification, and bioactivity assessment
Source: Front Nutr. 2024 Jan 19;10:1279552. doi: 10.3389/fnut.2023.1279552 (PMC10875998; doi:10.3389/fnut.2023.1279552)
Supplement: Supplementary file 1 [file Data_Sheet_1.docx]

Supplementary Material

**Supplementary Table 1.** MICs of vanillin standard against various pathogenic bacteria.

| **Concentration (mg/ml)** | ***Escherichia coli*** | ***Salmonella paratyphi*** | ***Shigella sonnei*** | ***Vibrio parahaemolytieus*** | ***Listeria monocytogenes*** | ***Staphyloccocus aureus*** |
| --- | --- | --- | --- | --- | --- | --- |
| 2 | + | + | + | + | + | + |
| 4 | + | + | + | - | + | + |
| 6 | + | - | - | - | - | - |
| 8 | - | - | - | - | - | - |
| 10 | - | - | - | - | - | - |

Note: Minimum inhibitory concentrations (MICs), of vanillin (standard) for each strain was determined by Microtitre Broth Dilution Method in a 96-well microtiter plate, OD values were measured using a microplate reader. In the negative controls, 100 μL of inoculum and 100 μL of LB medium were added to measure the normal growth of the microorganism. 200 μL of Lb medium without vanillin and bacterial inoculum was used as positive control. Plates were incubated at 37 °C for 18 hours. +; no growth inhibition at this concentration, -; growth inhibition.


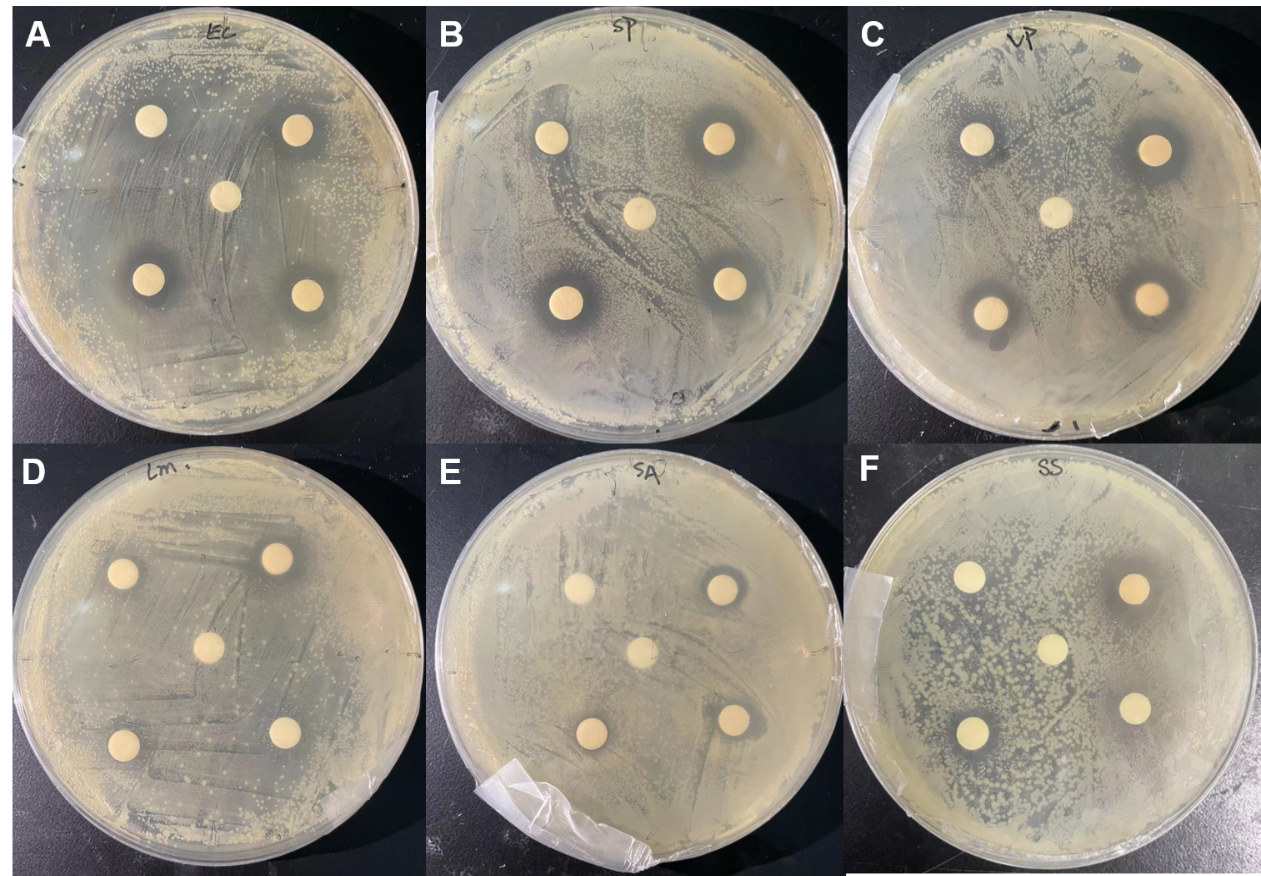


VS

VL

VP

VB

C

**Supplementary Figure 1.** Zone of inhibitions of various types of vanillin against food borne pathogens. A; *Escherichia coli*, B; *Salmonella paratyphi*, C; *Shigella sonnei*, D; *Vibrio parahaemolytieus*, E; *Listeria monocytogenes*, F; *Staphyloccocus aureus*. VS; vanillin standard, VP; vanillin from vanilla pod, VB; vanillin from bacterial fermentation broth, VL; vanillin from lignin and C; solvent as negative control.

AA
